# The Extent to Which Obesity and Population Nutrition Are Considered by Institutional Investors Engaged in Responsible Investment in Australia - A Review of Policies and Commitments

**DOI:** 10.3389/fpsyg.2020.577816

**Published:** 2020-12-23

**Authors:** Ella Robinson, Christine Parker, Rachel Carey, Gary Sacks

**Affiliations:** ^1^Institute for Health Transformation, Global Obesity Centre (GLOBE), Deakin University, Geelong, VIC, Australia; ^2^Melbourne Law School, The University of Melbourne, Melbourne, VIC, Australia; ^3^School of Agriculture and Food, The University of Melbourne, Melbourne, VIC, Australia

**Keywords:** responsible investment, obesity prevention, population nutrition, corporate accountability, corporate practices, sustainable finance

## Abstract

**Introduction:**

Responsible investment (RI), in which environmental, social and governance (ESG) considerations are incorporated into investment decision making, is a potentially powerful tool for increasing corporate accountability and improving corporate practices to address broad societal challenges. Whilst the RI sector is growing, there is limited understanding of the extent to which pressing social issues, such as obesity and unhealthy population diets, are incorporated within RI decision making. This study aimed to investigate the extent to which obesity prevention and population nutrition are considered by Australian institutional investors engaged in responsible investment.

**Methods:**

A desk-based review was conducted of investment approaches of prominent Australian asset managers and superannuation funds identified as engaged in responsible investment. Relevant information on the incorporation of ESG issues related to obesity and population nutrition was extracted for each investor, drawing on websites, published policy documents and annual reports. Strategies were categorized as: (1) negative/exclusionary screening; (2) positive/best-in-class screening; (3) norms-based screening; (4) ESG integration; (5) sustainability-themed investing; (6) impact/community investing; and (7) corporate engagement and shareholder action. These strategies were compared across investors and by themes related to obesity and population nutrition.

**Results:**

Eighteen of the 35 investors indicated that they applied investment strategies that considered issues related to obesity and population nutrition. The most commonly identified strategy was ESG integration (*n* = 12), followed by sustainability-themed investing (*n* = 6), and positive screening (*n* = 4). The ways in which obesity and population nutrition were considered as part of these approaches included relatively high-level general health considerations (*n* = 12), considerations around the healthiness of food company product portfolios (*n* = 10), and consideration of specific company nutrition policies and practices (*n* = 4). The specificity and depth to which RI strategies were disclosed varied.

**Conclusion:**

There is significant potential for investment decisions to contribute to efforts to address key social issues, such as obesity and unhealthy diets. Some institutional investors in Australia have recognized the potential importance of incorporating obesity- and population nutrition-related issues into decision-making processes. However, the extent to which these considerations translate into investment decisions and their impact on companies in the food sector warrant further exploration.

## Introduction

Unhealthy diets and dietary risk factors are the leading contributor to poor health worldwide (Afshin et al., 2019). The main drivers of unhealthy diets are unhealthy food environments, which are dominated by the supply, distribution and marketing of processed, packaged foods that are often high in salt, sugar, saturated fat and energy (Swinburn et al., 2011). In Australia, 8.4% of the burden of disease is specifically associated with overweight and obesity, and 7.3% associated with dietary risks (Australian Institute of Health and Welfare, 2019a). Two in three Australian adults and one in four Australian children and adolescents are now living with overweight and obesity (Australian Institute of Health and Welfare, 2019b). Australians are not consuming diets in line with dietary guidelines, with only 1 in 10 Australian adults consuming enough vegetables, and one third of energy in the Australian diet coming from unhealthy foods and beverages (Australian Institute of Health and Welfare, 2018). This has wide ranging impacts for not only health, but also the economy. Overweight and obesity has been estimated to cost the Australian economy AUD$8.6 billion between 2011 and 2012 in direct and indirect costs (not including reduced wellbeing and forgone earnings), primarily through loss in productivity, health systems costs and carer costs (Australian Institute of Health and Welfare, 2017). Considering the extensive health and economic costs, overweight and obesity presents one of the most pressing social issues facing the Australian population.

Global recommendations have identified the importance of a comprehensive response to improving the healthiness of food environments, including government policy, action from the food and agricultural industries, and broader societal change (World Health Organization, 2004, 2013; Swinburn et al., 2019). However, governments globally have been slow to develop and implement recommended policies and actions for addressing food environments, often due to a lack of political leadership as well as strong opposition from food industry actors (Mozaffarian et al., 2018; Swinburn et al., 2019). This is also true in Australia, with recent monitoring (conducted in 2017, 2019) of government action for healthy food environments finding that governments in Australia vary considerably in their implementation of internationally recommended policies, with a lack of comprehensive action (Sacks et al., 2017, 2019a). Additionally, recent evaluation of 27 actions to address obesity through the Australian National Preventive Health Taskforce found that in 10 years, only one action had been completed, 20 had progressed to a limited extent, and six had not progressed at all (Obesity Policy Coalition, 2020). Given the lack of government action to prevent obesity and related non-communicable disease in Australia and globally, it is especially important to investigate additional avenues that can contribute to a meaningful shift toward healthier food environments.

The financial sector is one such avenue that has significant potential to be a vehicle for change. As environmental and social challenges increase globally, the financial sector can play an integral role in driving a more sustainable and resilient society by considering stakeholder interests more broadly (UNEP Finance Initiative, 2017; Schoenmaker and Schramade, 2019). Whilst the traditional view on the purpose of financial agents and markets was to ensure profit maximization for shareholders, this approach is now being recognized as unsustainable and undesirable by global financial sector initiatives focused on sustainable development, such as the United Nations (UN) Environment Programme Finance Initiative and the UN Principles of Responsible Investment (Sandberg, 2015; United Nations Principles for Responsible Investment, 2020). Leading statements of good corporate governance are also recognizing the need to reconsider whether the purpose of the corporation is purely to maximize profit. In 2019 the Business Roundtable, which represents the Chief Executive Officers of leading companies in the United States, issued a new “Statement on the Purpose of a Corporation” which redefined the role of the corporation to deliver value for all stakeholders (Business Roundtable, 2019). This was a significant shift from previous Principles of Corporate Governance issued by the Business Roundtable, which maintained the traditional view that corporations exist primarily to serve their shareholders (Grove et al., 2020).

The concept of “sustainable finance” was borne out of the need for a revised global economic model, and can be defined as the integration of environmental, social and governance (ESG) criteria within financial decision making (Schoenmaker and Schramade, 2019). Sustainable finance can be viewed as an approach to finance that seeks to manage ESG-related risks, create long-term value and align decision-making with environmental, economic and societal goals, such those expressed through international agreements (European Commission, 2020b). The launch of the United Nations Sustainable Development Goals (SDGs) in 2015 provided a global framework for achieving sustainable societal and economic goals, and now presents a roadmap for all of society, including the financial sector, to address social and environmental issues (United Nations, 2015). At a government level, the European Union (EU) is arguably leading the way in sustainable finance, with the launch of the EU Green Deal, a roadmap for achieving climate neutrality in Europe by 2050 and the mobilization of <€1 trillion in sustainable investments over 10 years (European Commission, 2019, 2020a). In Australia, there have been some steps made toward a sustainable financial system through the Australian Sustainable Finance Initiative (ASFI). The ASFI aims to deliver a Sustainable Finance Roadmap for Australia to “support greater social, environmental and economic outcomes for the country” (Australian Sustainable Finance Initiative, 2019).

Investors are particularly well positioned to support and grow sustainable companies, industries and projects, as well as influence corporate behavior and governance through investment decision-making and shareholder actions (Thompson and Davis, 1997; McLaren, 2004; Oh et al., 2013). There has been a substantial rise in “responsible investment,” which incorporates ESG considerations into investment decisions (Wagemans et al., 2013; Global Sustainable Investment Alliance, 2018). The UN Principles of Responsible Investment (UNPRI) is one of the leading organizations promoting responsible investment, and over 3000 signatories have committed to a set of six voluntary principles aimed at contributing to a more sustainable global financial system (United Nations Principles for Responsible Investment, 2020). In 2018, according to the Global Sustainable Investment Alliance (GSIA), responsible investment assets across five major markets (Europe, United States, Canada, Japan, and Australasia) were equivalent to $30.7 trillion (Global Sustainable Investment Alliance, 2018). In Australia, the Responsible Investment Association of Australasia (RIAA) reported that responsible investors managed 44% of professionally managed assets in 2018, up 13% from the previous year (Thompson and Bayes, 2019).

Institutional investors [specialized financial institutions which collect funds from third parties to invest on their behalf (OECD, 2011)] have a high level of influence as they control significant assets (European Centre for Corporate Engagement, 2012). Through their diversified portfolios and longer term investments, institutional investors can be seen as reliant on a healthy economy for long-term positive financial performance (Dourma et al., 2017). Accordingly, efforts to improve economic and societal sustainability are important to their long-term and ongoing success. Institutional investor motivations for responsible investment thus often reflect financial considerations (to increase financial performance and ensure sustainable profit growth) as well as non-financial considerations (to reflect shareholder and stakeholder values) (Wagemans et al., 2013). Responsible investment can include strategies such as screening to exclude or include certain industries/companies, integration of ESG considerations into investment decision-making, and direct engagement with companies and shareholder action to address ESG considerations (Michael and Jacob, 2011; Eurosif, 2018; Global Sustainable Investment Alliance, 2018; Thompson and Bayes, 2019). These strategies can act in different ways to achieve different outcomes. For example, exclusionary screening is often focused on aligning portfolios with investor values; whereas positive screening and ESG integration are typically used to improve financial performance and achieve positive ESG-related outcomes (Kumar et al., 2016). Company engagement and shareholder action strategies primarily aim to influence company practices and behavior to generate long-term value (Kumar et al., 2016).

Whilst conflicting evidence exists, there is a growing body of research that indicates that responsible investment can have an impact on the ESG performance and ESG related disclosure and transparency of companies (Mackenzie et al., 2013; Eccles et al., 2017; Dyck et al., 2019). According to KPMG, approximately 71 percent of companies (out of 4,100) surveyed globally in 2013 now publish sustainability and Corporate Social Responsibility reports (KPMG International, 2013). Additionally, companies are increasingly reporting on ESG-related metrics through industry-led global reporting initiatives and assessments (Weber, 2018). Leading initiatives include the Global Reporting Index (GRI) (Global Reporting Initiative, 2018), and the SAM Corporate Sustainability Assessment, which forms the basis of the Dow Jones Sustainability Index and the S&P ESG Index family (RobecoSAM, 2019; S&P Global Ratings, 2020). Another global reporting initiative, the Task Force on Climate-related Financial Disclosures (TCFD), has developed recommendations on climate-related financial disclosures that can be used by both financial sector and non-financial sector organizations to assess climate related risks and opportunities (Task Force on Climate-Related Financial Disclosures, 2017, 2019). In Australia, regulatory disclosure rules also exist, with Recommendation 7.4 of the ASX Corporate Governance Council’s *Corporate Governance Principles and Recommendations* stating that listed companies must disclose material exposure to economic, environmental and social sustainability risks as well as how they manage (or intend to manage) those risks (ASX Corporate Governance Council, 2019).

Food sector companies are exposed to a number of risks related to obesity and unhealthy population diets. Regulatory risks are increasing for many companies in the food sector as a number of governments globally move to introduce policies such as: restrictions on the marketing of unhealthy foods and beverages (Global Food Research Program, 2020); front-of-pack labeling and warning labels on products high in salt, sugar and fat (Reyes et al., 2019; Taillie et al., 2020); and fiscal policies such as taxes of sugar-sweetened beverages (Colchero et al., 2017; Backholer et al., 2018). Reputational risks also exist, with increasing pressure on food sector companies (e.g., through public health and consumer advocacy groups) to better address health-related issues, and public exposure of the tactics employed by food and beverage companies to undermine public health (Ethical Investment Research Services, 2006). Additionally, consumers are increasingly informed about the negative health and planetary impacts of unhealthy foods and beverages, which is likely to present a risk to food sector companies that derive sales from these types of products (Access to Nutrition Initiative, 2020).

Several initiatives focus on engaging investors as part of a transition to a healthier food environment. ShareAction, a United Kingdom (UK) based charity, leads a campaign to mobilize institutional investment to improve the practices of food and beverage retailers and manufacturers in the UK (ShareAction, 2020). Similarly, the Access to Nutrition Initiative (ATNI) – a global benchmarking initiative that assesses prominent food and beverage companies on their policies and practices with regards to nutrition – has a large body of work dedicated to engaging with investors around the need to improve the policies and practices of major food and beverage manufacturers (Access to Nutrition Initiative, 2020). The “Farm to Fork” strategy as part of the EU Green Deal also aims to facilitate sustainable investments that support a transition to a healthy and sustainable food system, including actions to address food labeling, availability and affordability of healthy food and tax incentives (European Union, 2020). Whilst there are a number of initiatives in this area, there is limited academic research on how investors consider ESG issues related to obesity and population nutrition. Clapp and colleagues 2019 have explored how investment in agriculture and food (agri-food) businesses by large scale asset managers (e.g., BlackRock, Vanguard) is leading to increased corporate power and concentration amongst these businesses (Clapp, 2019). They show that a small number of large-scale asset managers hold significant investments in the same major agri-food businesses (common ownership), which leads to anti-competitive behavior and has negative implications for the sustainability and governance of food systems (Clapp, 2019). A previous study by Sacks and Robinson (2018) investigated existing mechanisms by which the investment sector could contribute to obesity prevention. The study found that whilst these issues were incorporated within the disclosure requirements and assessment indicators for initiatives that have widespread use amongst investors, such as the GRI, they were limited in scope and made up only a minor component of such initiatives (Sacks and Robinson, 2018). To the authors knowledge, there is no other academic literature exploring the extent to which issues related to obesity and population nutrition are considered within investment decision making and the investment strategies being used by investors to incorporate these issues.

This study aimed to make a contribution to addressing the identified knowledge gaps by: (i) investigating the extent to which ESG issues related to obesity and population nutrition are considered by institutional investors engaged in responsible investment in Australia; (ii) identifying the investment strategies declared in relation to the consideration of these issues; and (iii) exploring the specific obesity- and population nutrition-related themes investors use to guide their decision making.

This study adopted an interdisciplinary perspective to understand how a key social issue (i.e., obesity and population nutrition) is framed and addressed as part of the “S” in ESG by a sample of investors that are likely to broadly consider societal issues. This study contributes to the public health literature by exploring innovative avenues for addressing obesity and achieving population health goals. With respect to the business and finance literature, the study helps to shed light on an ESG issue that has received relatively little attention in Australia but is of significance given the societal costs and long-term risks that obesity and unhealthy population diets present to investors. From an organizational psychology perspective, the study contributes to the literature around advancing “social purpose” in organizations (Aguilera et al., 2007; Aguinis, 2011), in this case via increased attention to societal health issues within companies in the investment sector and the food industry.

The following part of this paper sets out the methodology for the desk-based review. The third part presents the results. The fourth part discusses the results and draws some conclusions around the implications for investors, the public health community and potential areas for further research and action.

## Methods

This study involved a desk-based review of a sample of asset managers and superannuation funds operating in Australia that are engaged in responsible investment. A desk-based review approach is consistent with a number of other initiatives that assess corporate practice and performance in regard to food system issues on the basis of publicly available information [such as the Business Benchmark on Animal Welfare (Amos et al., 2018), the Coller FAIRR Protein Producer Index (The FAIRR Initiative, 2019) and Plating up Progress (Nicholson, 2019)]. Details of each step of the desk-based review are outlined below.

### Identifying Asset Managers and Superannuation Funds for Inclusion in the Review

The most prominent asset managers and superannuation funds operating in Australia that are engaged in responsible investment were identified from recently published (2019) reports by the RIAA. The RIAA is the leading industry association for responsible investment in Australia and releases yearly reports that monitor and benchmark the size and ESG performance of Australian responsible investment asset managers and superannuation funds. The sample of 35 asset managers and superannuation funds included in this study was drawn from these benchmarking reports (further details provided below) and can be considered a best practice sample of responsible investors in Australia.

First, the sample included the 21^1^ Australian asset managers identified by RIAA as “applying a leading approach to ESG integration” in the 2019 RIAA Responsible Investment Benchmark Report (Thompson and Bayes, 2019). “Leading approach” was defined by the RIAA as having scored over 80% on the RIAA ESG integration assessment. This assessment was based on a desktop review, conducted by RIAA, of each investment manager against a framework of leading practice in ESG integration. Only 28% of those investment managers assessed by RIAA were included in this leading group, and this, therefore, represents a best practice group in Australia, as judged by the leading industry association.

Second, the sample included three additional asset managers that were identified in the 2018^2^ RIAA Responsible Investment Benchmark Report as the largest asset managers using responsible investment strategies other than ESG integration (Responsible Investment Association Australasia, 2018). These other strategies include negative and positive screening, norms-based screening, sustainability-themed investing, impact/community investing, and corporate engagement and shareholder action. Largest in this context refers to the amount of assets under management. The other 11 largest asset managers identified by the RIAA as using these other strategies had all already been included in our sample (see above).

Third, the sample included the 12^3^ Australian superannuation funds classified as “leading” funds in the 2019 RIAA Responsible Investment Super Study (Boele et al., 2019)^4^. The 2019 RIAA Responsible Investment Super Study defined the “leading” superannuation funds as the top 25% of all superannuation funds assessed with reference to RIAA’s “Framework of Good Responsible Investment Governance” and a scale “indicating the quality and scope of disclosures” (Boele et al., 2019). Asset managers and superannuation funds were only included if they were headquartered in Australia or identified as Australian investors by RIAA.

### Data Collection

Searches of publicly available information were conducted (up until December 2019) relating to the responsible investment approaches of the institutional investors included in the study. First, a general search of the websites of each institutional investor was conducted to identify pages related to the investor’s responsible investment strategies, policies or approaches. Relevant webpages were then searched using key terms related to obesity and public health nutrition, refined based on a preliminary assessment of content on investors’ websites. Terms included: obesity, nutrition, health, diet, disease, food, soft drink, sugar, beverage. Additionally, a search of publicly available data in the 2019 UNPRI transparency reports was conducted, using the same key terms. Signatories to the UNPRI report on their responsible investment activities each year through these transparency reports.

References to “health and safety,” “occupational health,” or “healthcare” were excluded. References to food sustainability-related issues (including food scarcity) were also excluded and are the subject of a separate study. Relevant documents, articles and webpages were downloaded, or images captured.

### Content Analysis

A content analysis of the data collected from websites and documents was conducted using Microsoft Word. Content analysis includes a range of approaches to analyzing text data in order to understand the research phenomena under enquiry (Nandy and Sarvela, 1997; Hsieh and Shannon, 2005). In this case, a “directed content analysis” approach was adopted, in which prior research was used to develop an initial coding scheme as part of a deductive analysis – described further below (Hsieh and Shannon, 2005).

Where investors referenced relevant obesity and nutrition-related key words, the responsible investment strategy to which it related was identified. Identified strategies of responsible investment were coded based on the RIAA’s definitions, which also align closely with definitions used by other responsible investment industry associations, such as the European Sustainable Investment Forum and the Global Sustainable Investment Alliance. These strategies included: (1) negative/exclusionary screening; (2) positive/best-in-class screening; (3) norms-based screening; (4) ESG integration; (5) sustainability–themed investing; (6) impact/community investing; and (7) corporate engagement and shareholder action. See Table 1 for information on these responsible investment strategies and their definitions. A set of criteria was developed to assist in coding the different strategies. Refer to the Supplementary Table 1 for details of the criteria for coding of strategies.

**TABLE 1:** Responsible investment strategies and definitions.

Responsible investment strategy	Definition^1^
(1) Negative/exclusionary screening	Screening that systematically excludes specific sectors, companies or practices based on ESG criteria. Common criteria used in negative screening include gambling, alcohol, tobacco, weapons, pornography and animal testing.
(2) Positive/best-in-class screening	Involves the inclusion of sectors, companies or projects based on positive ESG or sustainability performance relative to industry peers. It involves identifying companies with superior ESG performance from a variety of industries and markets.
(3) Norms-based screening	Screening of investments that do not meet minimum standards of business practice, based on international norms and conventions (e.g., those from the United Nations). This often results in the exclusion of assets that contravene these norms and conventions, or inclusion of assets that uphold them.
(4) ESG integration	ESG integration involves the systematic and explicit inclusion of environmental, social and governance factors into traditional financial analysis and investment decision-making by investment managers.
(5) Sustainability-themed investing	Investment in themes or assets that specifically relate to improving social and environmental sustainability. This commonly involves funds that invest in clean energy, green technology, sustainable agriculture and forestry, green property or water technology.
(6) Impact and community investing	Impact investing included targeted investments aimed at addressing social or environmental issues whilst also creating positive financial returns. Community investing includes investment in underserved individuals or communities, as well as businesses with a social and environmental purpose.
(7) Corporate engagement and shareholder action	The employment of shareholder power to influence corporate behavior in relation to ESG issues. This may be conducted through direct corporate engagement such as communications with senior management or boards, filing or co-filing shareholder proposals and proxy voting.

*^1^Adapted from the Responsible Investment Association of Australasia (RIAA), Responsible Investment Benchmark Report 2019 (Thompson and Bayes, 2019).*

The approach noted by each investor was then coded based on the way in which issues relevant to obesity and population nutrition were considered. This process was initially done deductively, with the coding framework drawn from a food environment monitoring framework developed by the International Network for Food and Obesity/NCD Research, Monitoring and Action Support (INFORMAS) (Swinburn et al., 2013)^5^. These initial codes included food composition, food labeling, food marketing, food provision, food retail, food prices and food trade and investment (Swinburn et al., 2013). Data that did not apply to the initial codes were subsequently analyzed inductively to identify additional codes. All the coded data were then reviewed and compared, with data grouped together by various themes related to obesity and population nutrition in an iterative process. The themes were: (1) general health considerations relevant to obesity and population nutrition; (2) company nutrition policies and practices; and (3) company product portfolio. “General health considerations relevant to obesity and population nutrition” included broadly defined investment considerations related to health, obesity and nutrition, and/or Sustainable Development Goals 2 and 3 (where nutrition, obesity or health is specifically mentioned). “Company nutrition policies and practices” included investment considerations and/or engagement activities related to the policies and practices of companies in the food industry directly related to obesity and population nutrition, including: food marketing; food reformulation and product development; and disclosure and transparency around relationships with external groups. “Company product portfolio” included investment considerations related to the “healthiness” of the product portfolio of companies in the food industry. Full details of the themes and sub-themes are outlined in the Supplementary Table 2.

All coding and thematic analysis were initially performed by one coder (ER) and then were cross-checked by a second coder (CP). Any discrepancies in agreement between the two coders were then discussed and resolved by the authorship team.

## Results

### Investors Included in the Sample

A total of 35 institutional investors were included in the sample (Table 2). Twenty-four were asset managers, and twelve were superannuation funds^6^. Amongst the superannuation funds, two were retail funds (for profit, owned by financial institutions, offered to the public), six were industry funds (not for profit, traditionally focused on a single industry, often co-owned by employers and unions in that industry), and four were public/non-regulated funds (not for profit, established for government sector employees, generally only available to public sector employees). Fourteen investors had <$10 billion assets under management (AUM), 15 had between $10 and $100 billion AUM, and five had >$100 billion AUM. Two investors (RealIndex Investments and Stewart Investors) were subsidiaries of First Sentier Investors at the time and as such their AUM were included within First Sentier Investors.

**TABLE 2:** Characteristics of asset managers and superannuation funds included in the sample, as at 2019.

Asset managers	Total assets under management (AUD billion)^1^	Superannuation funds	Total assets under management (AUD billion)^1^	Type of fund and broad focus^2^
AMP Capital	$187.25	Australian Ethical	$2.82	Retail – Ethical focus
Ausbil Investment Management	$10.48	Australian Super	$142.19	Industry – Default fund for all industries (largest super fund in Australia)
Australian Ethical Investment	$2.82	Care Super	$14.76	Industry – Professional and service industries
Dexus Property Group	$27.20	Cbus	$46.69	Industry – Building and construction
First Sentier Investors	$204.19	Christian Super	$1.42	Industry – Christian community schools
IFM Investors	$117.09	First State Super	$70.56	Public/non-regulated – originally for NSW Government employees, later merged with health and teaching funds
Investa Property Group	$11.00	Future Super	$0.34^3^	Retail – Environmental sustainability focus
Lendlease Investment Management	$33.00	HESTA	$46.87	Industry – Health and community services
Magellan Asset Management	$45.50	Local Government Super	$7.79	Public/non-regulated – Local government employees
Maple-Brown Abbot	$14.34	UniSuper	$70.54	Industry – Higher education and research
Mercer Australia	*n/a*	VicSuper^5^	$21.21	Public/non-regulated – Public sector
Pendal	$92.82	Vision Super	$9.65	Public/non-regulated – Community sector
Pengana Capital Group	$3.01			
Perpetual Investments	$30.80			
QIC	$85.01			
RARE Infrastructure	$3.53			
Realindex Investments^5^	*n/a*			
Resolution Capital	$7.78			
Solaris Investment Management	$7.61			
Stafford Capital Partners	$4.86			
Stewart Investors^4^	*n/a*			
U Ethical Funds Management	$1.12			
Uniting Financial Services	$1.63			
Wavestone Capital	$3.90			

*^1^As at 2018. Sourced from United Nations Principles of Responsible Investment. Transparency reports (2019), unless otherwise indicated (United Nations Principles for Responsible Investment, 2019). Total assets under management includes subsidiaries and excludes advisory/execution only assets. ^2^Retail fund = for profit, owned by financial institutions, offered to the public; Industry fund = not for profit, traditionally focused on a single industry, often co-owned by employers and unions in that industry; Public/non-regulated = not for profit, established for government sector employees, generally only available to public sector employees. ^3^As at June 2018. Sourced from the Future Super. Annual Report, 2018 (Future Super, 2018). ^4^Wholly owned subsidiary of First Sentier Investors. ^5^VicSuper merged with First State Super as of July 01, 2020. n/a = Information not available.*

### Strategies Used by Investors to Incorporate Obesity and Population Nutrition Issues Into Investment Decision Making

Table 3 provides an overview of strategies used by investors to incorporate obesity and population nutrition issues into investment decision making. A total of 18 asset managers and superannuation funds used responsible investment strategies that related to obesity and population nutrition. The most commonly identified strategy was ESG integration (*n* = 12), followed by sustainability-themed investment (*n* = 6), positive/best-in-class screening (*n* = 4), corporate engagement and shareholder action (*n* = 3) and negative/exclusionary screening (*n* = 2). No investors were shown to use norms-based screening or impact/community investing strategies. Twelve investors used strategies that involved the theme of “general health considerations relevant to obesity and population nutrition,” of which the majority were ESG integration and sustainability-themed investment strategies. Ten investors used strategies that involved the “company product portfolio” theme – these primarily related to an ESG integration investment strategy. Only four investors used strategies involving the theme of “company nutrition policies and practices,” and unlike the other themes, these strategies almost exclusively involved corporate engagement and shareholder action. For a summary of the relevant investment strategies disclosed by investors, refer to Supplementary Table 3. For in-depth details on investment strategies of asset managers and superannuation funds and obesity/population nutrition related themes addressed, refer to the Supplementary Table 4.

**TABLE 3:** Investment strategies of asset managers and superannuation funds and the obesity/population nutrition theme to which they relate.

Investment strategy	Theme related to obesity and population nutrition		
	General health considerations relevant to obesity and population nutrition^1^	Company nutrition policies and practices^2^	Company product portfolio^3^
Negative/exclusionary screening	• Australian Ethical		• Australian Ethical • Christian Super
Positive/best-in-class screening	• Pendal • U Ethical		• Australian Ethical • Future Super
Norms-based screening			
ESG integration	• HESTA • Magellan Asset Management • Mercer Australia • Pendal • Perpetual Investments • Stafford Capital Partners	• Christian Super	• AMP Capital • Ausbil Investment Management • Australian Ethical • Christian Super • First Sentier Investors • Magellan Asset Management • Pendal • Stewart Investors
Sustainability-themed investing	• CareSuper • First Sentier Investors • Pengana Capital • U Ethical • Uniting Financial Services		• Pengana Capital • Stewart Investors
Impact/community investing			
Corporate engagement and shareholder action		• AMP Capital • Local Government Super • Stewart Investors	• AMP Capital
*No investment strategy identified in relation to obesity and population nutrition themes*	Australian Super, Cbus, Dexus Property Group, First State Super, IFM Investors, Investa Property Group, Lendlease Investment Management, Maple-Brown Abbot, QIC, RARE Infrastructure, Realindex Investments, Resolution Capital, Solaris Investment Management, UniSuper, VicSuper, Vision Super, Wavestone Capital.		

*^1^Includes broadly defined investment considerations related to health, obesity and nutrition, and/or Sustainable Development Goals 2 and 3. ^2^Includes investment considerations and/or engagement activities related to the policies and practices of companies in the food industry directly related to obesity and population nutrition, including: food marketing; food reformulation and product development; and disclosure and transparency around relationships with external groups. ^3^Includes investment considerations related to the “healthiness” of the product portfolio of companies in the food industry. ESG = environmental, social and governance.*

#### Negative/Exclusionary Screening

Two investors reported using a negative/exclusionary screening strategy. Australian Ethical, an ethically focused fund that only invests in businesses that align with their Ethical Charter, screened investments based on their positive and negative impacts on people. They avoided any investment in products, goods or services that have a harmful effect on humans. Australian Ethical specifically stated that they avoid investment in food producers and food products that do not align with a healthy diet. This included producers of foods that were “overconsumed (e.g., sugar)” and food products high in *trans*-fat, sugar and salt. Notably, Australian Ethical referred to using World Health Organization guidelines to assess the nutritional quality of products. Christian Super, a Christian-values based industry fund originally formed for teachers in Christian community schools, specified that they would avoid investments in companies that produced addictive or harmful goods and services, which explicitly included the fast food industry. Both Christian Super and Australian Ethical were ethically focused smaller investors (<$3 billion AUM).

#### Positive/Best-in-Class Screening

Four investors used a positive screening strategy. The positive screening approach of two investors, Pendal and U Ethical, were classified as relating to the “general health considerations relevant to obesity and population” theme. Pendal reported using positive screens that included industries with products and services that “improved health and community wellbeing.” Similarly, U Ethical, a Christian-values based asset manager that is an autonomous enterprise of the Uniting Church, used a positive screening approach that encouraged investment in companies that produced goods or services that enhanced the health and welfare of individuals and communities.

Two investors reported positive screening approaches that were based on companies or products that were considered “healthy.” Future Super, a climate change focused fund set up by activists, had a positive screening process that sought out companies involved in the production of healthy foods and support for healthy lifestyles. Australian Ethical stated that, as part of their “Ethical Charter,” they would positively screen companies involved in the production of healthy food:

*“Food must be nutritious before we can invest in its production. In assessing whether a food can form part of a balanced and healthy diet we take into account credible sources like the World Health Organisation’s (WHO) healthy diet guidelines. In practice, this means we are unlikely to invest in producers of food that is overconsumed* (e.g., *sugar*) *and look more favorably on producers of fruits, vegetables, legumes, nuts and whole grains.”* – Australian Ethical (Australian Ethical, 2019).

Both Australian Ethical (the superannuation arm) and Future Super were smaller retail funds with an ethical focus (<$3 billion AUM).

#### Norms-Based Screening

No norms-based screening strategies were identified in relation to obesity and population nutrition related themes.

#### ESG Integration

Environmental, social and governance integration was the most commonly identified of the investment strategies, with a total of 12 investors applying this strategy in relation to obesity and population nutrition themes.

Environmental, social and governance integration was primarily seen in relation to the “company product portfolio” theme, with eight investors reporting considerations related to the “healthiness” of a company or product. This predominantly centered around companies or products that were “unhealthy,” and to a lesser extent companies or products that were “healthy.” Christian Super reported that they would review major food producers on their product range and would exclude those that performed below industry standards. AMP Capital, Ausbil Investment Management, First Sentier Investors, Magellan Asset Management, Pendal and Stewart Investors (all relatively large asset managers, > $40 billion AUM) all raised concerns around unhealthy food sector companies and products being exposed to financial risks due to the public health issue of obesity. For example, the threat of government regulation (e.g., through a sugar-sweetened beverage tax) and changes to consumer preferences (e.g., shifts to healthier or “wellness” products) were seen as posing a risk to earnings for exposed companies, particularly sugar-sweetened beverage or snack food manufacturers.

“*The* “*obesity*” *theme is well documented and presents both opportunities and risks for investors*…. *there are food & beverage companies that are negatively exposed and with limited possibility for adaptation to changing consumer and regulatory trends, for which it is hard to see an easy transition”* – Ausbil Investment Management (Ausbil Investment Management, 2016).

Investors’ responses to considering obesity-related risks varied, with the majority, including AMP Capital, Ausbil Investment Management, First Sentier Investors and Magellan Asset Management flagging this as an area for investors to monitor. Stewart Investors, a boutique stewardship focused firm whose fund management approach aims to benefit all stakeholders and the wider community, Pendal and Christian Super, explicitly stated that they had sold out of or reduced investment in some exposed companies.

*“Coca Cola Amatil (CCL) is one of Asia-Pacific’s largest bottlers and distributors of alcoholic and non-alcoholic beverages*…. *For many years, Pendal Group Limited (Pendal) has held concerns regarding headwinds from structural shifts in consumer demand for healthier options and regulatory risks relating to sugar consumption and their associated impacts on corporate profitability. Pendal has held an underweight position in CCL across its Australian fixed income funds for a number of years, given these concerns”* – Pendal Group (Pendal Group, 2018).

Stewart Investors and Australian Ethical also reported ESG integration activities in relation to companies or products that were “healthy.” Stewart Investors had invested in two companies that were focused on providing healthier products. Australian Ethical stated that they considered the health impacts of food produced when making investment decisions around agricultural activities and companies.

The ESG integration strategies of six investors were classified under the theme of “general health considerations relevant to obesity and population nutrition.” HESTA, an industry fund set up for the healthcare and community services sector, reported looking at the impact of investments on people and planet as part of their decision-making process, including considerations related to “good health and wellbeing.” Magellan Asset Management reported that companies that have a major detrimental impact on human health are high risk investments and warrant scrutiny by investors. Mercer Australia reported exploring ESG investment themes that included health, which was reflected in their investment advice, research and portfolio monitoring. Pendal noted a range of financially material ESG factors that were reviewed as part of the ESG integration process, including products or services that have positive impacts like improved health and community wellbeing and disease prevention. Perpetual Investments reported evaluating companies based on their performance in various ESG issues areas, one of which included obesity. Stafford Capital Partners reported assessing companies for alignment with the SDGs as part of their ESG integration approach, including SDG2: Zero Hunger and SDG3: Good Health and Wellbeing. Stafford Capital Partners is a boutique firm that specializes in real assets and reports that responsible investment is core to their long-term strategy.

Only one investor, Christian Super applied ESG integration in relation to the “company nutrition policies and practices” theme, specifically in relation to food marketing practices. Christian Super reported that they would review major food producers on their marketing practices and would exclude those that performed below industry standards. They did not specify which industry standards were being applied.

#### Sustainability-Themed Investing

Six investors explicitly identified sustainability-themed investments that considered obesity- and nutrition-related issues. Among those investments explicitly identified as sustainability-themed investments, the most frequently noted considerations related to the theme of “general health considerations relevant to obesity and population nutrition.” First Sentier Investors, Pengana Capital and Uniting Financial Services (a Christian-values based charitable investment service) all reported sustainability-themed investments that targeted companies with a positive impact on health. U Ethical reported sustainability-themed investments that supported the Sustainable Development Goals, specifically SDG2: Zero hunger, which had led to investment in two food companies (Unilever and the A2 Milk Company). Similarly, CareSuper reported choosing investments that aligned with the SDGs, specifically including SDG3: Good Health and Wellbeing. CareSuper also reported seeking out positive investments that included improved nutrition.

Two investors, Pengana Capital and Stewart Investors, reported sustainability-themed investments that related to the “company product portfolio” theme. Pengana Capital reported that their Sustainable Impact Fund would not invest in Kraft Heinz or Unilever due to those companies producing products that contribute to unhealthy lifestyles. Stewart Investors stated that their sustainable goods and services theme seeks out companies that provide food and beverages that are positive for human health. Stewart Investors also reported focusing their investments on companies that appreciate the risks associated with malnutrition and are working toward improving access to nutritious products.

*“The private sector, by virtue of its scope and pervasiveness, has a vital role to play in developing a sustainable solution to the problem of malnutrition. As such, we continue to aim to allocate clients’ capital to companies that we feel are beginning to realize the necessity improving access to nutritious products, and believe that this is beneficial both for society and long-term investment returns”* – Stewart Investors (Stewart Investors Sustainable Funds Group, 2018).

#### Impact/Community Investing

No impact/community investing strategies were identified in relation to obesity and nutrition.

#### Corporate Engagement and Shareholder Action

Only three investors reported using corporate engagement and shareholder action strategies that related to obesity and population nutrition issues. Unlike the other strategies, corporate engagement and shareholder action was almost exclusively in relation to the “company nutrition policies and practices” theme. AMP Capital, Local Government Super and Stewart Investors reported activities that related to food reformulation/product development policies and practices. AMP Capital noted that they were engaging with food and beverage companies on their progress to meet sugar reduction targets. Similarly, Stewart Investors reported that they continued to engage with a food company on the sugar content of their products. Local Government Super reported engaging with companies on how they were improving the nutritional benefits of their products. AMP Capital and Local Government Super also reported engaging with companies around generally improving policies and practices related to obesity and population nutrition and discussed how they were using the findings of the Access to Nutrition Initiative (ATNI) in their engagement with companies. Local Government Super was the only public/non-regulated superannuation fund to disclose investment strategies related to obesity and population nutrition. Both investors disclosed that they had asked companies to report on how they were integrating recommendations from the ATNI. AMP Capital went further in that they also reported meeting with the boards and management of food and beverage companies with the goal of reducing marketing to children and young adults and requesting disclosure on political donations and funding of scientific research.

*“AMP Capital fund managers have been meeting with the boards and management of Australia’s largest food and beverage manufacturers asking for reductions in sugar usage and changes to the way the companies advertise to children. AMP capital has been asking food and beverage companies to report on their progress meeting sugar reduction targets as well as for details of advertising policies and information about how they fund scientific research.”* – AMP Capital (AMP Capital, 2019).

AMP Capital was the only investor to report on corporate engagement and shareholder action activities in relation to the “company product portfolio” portfolio. They reported engaging with food and beverage companies on the need to diversify earnings streams. AMP Capital was one of the larger investors in the sample, with >$187 billion AUM.

## Discussion

This study involved a desk-based review of the extent to which Australian institutional investors engaged in responsible investment incorporate ESG issues related to obesity and population nutrition. Eighteen of the 35 investors included in the sample were identified as using responsible investment strategies to incorporate issues related to obesity and population nutrition. The findings indicate that the way in which obesity and population nutrition are considered varies substantially across a best practice sample of institutional investors in Australia. While issues related to obesity and population nutrition are discussed by some institutional investors as part of their approach to considering social issues within ESG, the ways in which these issues impact decision-making and the extent to which they are prioritized when compared to other environmental, social and governance issues is not clear.

### Responsible Investment Strategies Adopted by Investors to Address Obesity and Population Nutrition Related Issues

The most common strategy observed was ESG integration, which was usually in relation to the healthiness of a company’s product portfolio. A number of investors also reported on sustainability-themed investment activities, generally in relation to specific funds or investments that aimed to improve sustainability with regards to health. Some investors declared that they used positive screening approaches, and others reported using negative screening approaches. In most of these screening approaches, as with ESG integration activities, decisions were typically based on the healthiness of a company’s product portfolio. Interestingly, only a small number of investors were identified as using corporate engagement and shareholder action for concerns related to obesity and population nutrition. These investors reported that they engaged with food and beverage companies to improve policies and practices related to nutrition. Eurosif (2013) notes that corporate engagement and shareholder action is frequently combined with ESG integration and negative screening (Eurosif, 2013), and, as such, may not have been specifically reported against as part of an overall responsible investment strategy.

The findings from this study align with data from the Global Sustainable Investment Alliance (GSIA) and the RIAA which suggest that ESG integration is the dominant strategy used by the responsible investment community in Australia (Global Sustainable Investment Alliance, 2018; Thompson and Bayes, 2019). The comprehensiveness and specificity of ESG integration strategies varied significantly across investors, with some investors discussing obesity and population nutrition issues in terms of potential risk exposure, and others providing more detail on the actions they had taken as a result of factoring in obesity- and population nutrition-related ESG considerations into investment decision-making processes. It has been argued that ESG integration is largely in line with traditional financial analysis, expanding on financial factors that are included in the decision-making process to also include ESG, with the underlying motivation being higher returns and financial risk management (Parfitt, 2019). It is therefore not surprising that institutional investors in this study were observed engaging in a range of activities that fell under the category of “ESG integration,” as there is likely to be significant variability in what investors decide are financially material risks, and how these will affect the performance of an investment portfolio or company.

While a number of investors were identified as using responsible investment strategies to incorporate issues related to obesity and population nutrition within their investment decision-making, investors were not ranked based on their disclosed approaches to the topic. Australian Ethical and Stewart Investors were identified as disclosing the most investment strategies (three strategies) related to obesity and population nutrition. Five investors (AMP Capital, Christian Super, First Sentier Investors, Pendal, and U Ethical) disclosed two strategies, eleven investors disclosed one strategy and the remaining 17 investors did not disclose any investment strategies related to obesity and population nutrition. Future research should consider which responsible investment strategies are the most effective from a public health nutrition perspective. Furthermore, this study did not assess investors based on their approach to responsible investment more broadly or their reputation as a leader or laggard in responsible investment. For example, while AMP Capital (a subsidiary of AMP), whose corporate engagement and shareholder action approach aligned with ATNI recommendations, was criticized in the 2017-2019 Royal Banking Commission (Commonwealth of Australia, 2019), this was not incorporated into our analyses. Several superannuation funds, such as Cbus, First State Super, VicSuper and VisionSuper were recently recognized as leaders in responsible investment at a global level through the UNPRI Leaders’ Group 2019 (United Nations Principles of Responsible Investment, 2019), although they were not identified in this study as leaders in relation to obesity and population nutrition specifically. This indicates that obesity and population nutrition related issues are likely still emerging and not being comprehensively addressed, even by those investors that demonstrate best practice in responsible investment more broadly.

### Characteristics of Investors

Several investors that were identified as incorporating obesity and population nutrition related issues had a specific ethical focus (Australian Ethical, Future Super) and/or had a Christian values-based focus (Christian Super, U Ethical, Uniting Financial Services). Screening strategies were almost exclusively disclosed by investors that had an ethical or Christian values-based focus. Stewart Investors, a boutique firm that focuses on stewardship and responsible investment for long-term value creation, and Australian Ethical, disclosed the most responsible investment strategies related to obesity and population nutrition overall (*n* = 3). Investor motivations for considering obesity and population nutrition and ESG issues more broadly are likely to vary between investors, including predominantly financial motivations, motivations that are focused on responding to pressure from governments or the public and more ethically driven motivations (von Wallis and Klein, 2015). Considering the desk-based nature of this study, conclusions as to the motivations driving the consideration of obesity and population nutrition by different investors were not able to be drawn. The proportion of investors that disclosed approaches to considering obesity and population nutrition was similar across asset managers and superannuation funds (13/24 versus 6/12). Three of the superannuation funds were industry funds, two were retail funds (noting there were no other retail funds in the sample), and one was a public/non-regulated fund. There may be a number of additional investors that are taking action in this area but are not publicly reporting on it. Further research that engages directly with investors should investigate the barriers and enablers to consideration of these issues. This research could also investigate whether investors who are in the business of “health” (e.g., HESTA whose membership base is primarily the healthcare sector, or health insurance companies) might be more motivated to take the lead in this area.

The following sections discuss the obesity and population nutrition themes identified during this study, the need for comprehensive reporting and ESG data on obesity and population nutrition issues and what implications these findings have for the public health community.

### Obesity and Population Nutrition Themes Identified

#### General Health Considerations Relevant to Obesity and Population Nutrition

“General health considerations relevant to obesity and population nutrition” was the most common theme identified. This is unsurprising considering the broad nature of this theme, which included a wide range of non-specific considerations related to health, obesity and nutrition. The majority of strategies in relation to this theme were in regard to “health” broadly, with relevant strategies involving ESG integration related to the health of individuals and communities as well as sustainability-themed investments focused on health-related outcomes. However, it was typically unclear whether investors considered obesity and nutrition as part of general “health” considerations, and the extent to which general considerations around health translated into investment with regards to companies in the food sector was unclear.

A small number of investors specifically reported responsible investment activities that incorporated SDG2 “Zero Hunger” and SDG3 “Good Health and Wellbeing.” These two SDGs have clear links to obesity and population nutrition, and investment focus on SDG2 and SDG3 are likely to consider obesity and population nutrition issues to some extent. The 2017 Global Nutrition Report and the World Obesity Federation have noted that almost all of the SDGs can be linked to obesity and nutrition (Development Initiatives, 2017; Cooper, 2019). Despite this, obesity is not explicitly mentioned by the SDGs, with only one indicator out of 231 mentioning overweight specifically, and only in relation to children under 5 years of age (United Nations, 2015). The almost complete omission of overweight and obesity within the SDGs may present a roadblock for investment action in the area, particularly because of increased focus on achieving the SDGs by the investment community (Dourma et al., 2017).

#### Company Product Portfolio

In Australia, the RIAA reported in their 2019 Responsible Investment Benchmark Report that negative screening of “junk food” was on the rise, with 13% of respondents in 2018 reporting this as an issue being screened, up from 5% the previous year (Thompson and Bayes, 2019). However, few investors included in this review disclosed screening strategies based on a company’s product portfolio. The exceptions in this study were Australian Ethical, who stated that they would avoid investment in unhealthy foods, and Christian Super, who explicitly reported that they avoid investment in the fast food industry. The discrepancy in findings between this study and the RIAA benchmark report is likely due to RIAA using more detailed self-reported survey data (rather than publicly available) and surveying a larger sample of investors (*n* = 68) (Thompson and Bayes, 2019). Based on the findings from RIAA, there are likely to be a number of other institutional investors in Australia that use screening strategies based on the healthiness of company product portfolios. Future research should explore the extent to which investors screen companies based on the healthiness of their product portfolios through direct engagement with the investment community.

The primary strategy observed in relation to the company product portfolio theme was ESG integration. Taxation and the threat of government regulation were highlighted as growing financial risks to the food and beverage industry by several investors included in this review. These risks were noted particularly for sugar-sweetened beverage and unhealthy snack food manufacturers, but also in relation to food and beverage companies more generally as well as meat products. Furthermore, consumer wellness trends leading to decreases in consumption of “less healthy” products and increases in demand for “healthier” products were also recognized as a financial risk to the sector. These types of risks to earnings for the food sector have been noted previously in reports published by the investment sector. For example, in 2006, Vigeo-EIRIS (then EIRIS), an ESG ratings and research company, produced a report on the risks associated with obesity and how these were likely to emerge for food and beverage companies and fast food chains (Ethical Investment Research Services, 2006). Key risks identified in the Vigeo-EIRIS report aligned with those identified in this review, including regulatory changes (e.g., restrictions on marketing to children and mandatory labeling), legal risks (e.g., litigation from consumer advocacy and public health groups) and damaged brand reputation (e.g., through negative brand exposure). Vigeo-EIRIS also developed a risk exposure methodology based on a company’s relative and absolute turnover derived from the production or sale of “unhealthy” food and beverage products. More recently, Credit Suisse published a 2013 research report on the negative health impacts of sugar and associated risks for food and beverage companies (Credit Suisse Research Institute, 2013). As with the Vigeo-EIRIS report, Credit-Suisse highlighted the risks posed by regulation and taxation, as well as negative public opinion and consumer awareness of the, so called, sugar debate (Credit Suisse Research Institute, 2013). While these reports highlight obesity-related risks for companies and industries that are exposed, there are also significant investment opportunities, as noted by several investors included in this review, who had invested in food sector companies that produced healthier and more sustainable products.

Overall, the methods through which investors determine the healthiness of a company’s portfolio appear to be inconsistent. For example, UEthical reported investing in Unilever due to its provision of “nutrition” products; however, Pengana Capital reported that their sustainable impact fund would not invest in Unilever due to its unhealthy products brands (e.g., Ben and Jerry’s ice cream). As major food and beverage manufacturers, retailers and fast food companies often have diverse portfolios or product offerings, determining their overall “healthiness” and its associated risk exposure may prove difficult. Tools such as the Access to Nutrition Initiative are likely to be helpful for investors when making these decisions. The Access to Nutrition Initiative published a comprehensive product portfolio assessment of 21 major food and beverage manufacturers across nine markets (Access to Nutrition Initiative, 2017). This assessment provided each company’s product portfolio with a score (out of 10) based on the proportion of the company’s sales derived from products of different categories of “healthiness” (with a score of 10 meaning all sales are derived from the “healthiest” products, and a score of 0 meaning all sales are derived from the least healthy products). The methodology used the Australian Government endorsed Health Star Rating nutrient profiling system and the “WHO Europe Nutrient Profile model for marketing to children” (Access to Nutrition Initiative, 2017) for assessing product healthiness. Similarly, the George Institute for Global Health has conducted a product portfolio assessment of major food and beverage manufacturers and fast food companies in Australia which assigns a mean Health Star Rating (from 0.5 as the least healthy, to 5 as the most healthy) to a company’s portfolio or product offerings (Neal et al., 2019; Howes et al., 2020). These types of composite and easily understood assessments of the “healthiness” of food sector companies can provide investors with evidence-based tools to make investment decisions that incorporate company product portfolio considerations.

#### Nutrition Policies and Practices

The few observed examples of the “nutrition policies and practices” theme were primarily in relation to corporate engagement and shareholder action. This aligns with the common motivations behind corporate engagement and shareholder action, which are generally focused on influencing corporate practice and behavior for long term value creation (Kumar et al., 2016; McNulty and Nordberg, 2016). There are a small number of not for profit initiatives that focus on engaging investors to improve food industry policy and practice with regards to obesity and malnutrition. Initiating corporate engagement and shareholder action activities from investors is likely to be a key component of this approach. The most prominent global initiative is the aforementioned ATNI, which, alongside a product portfolio assessment, benchmarks major food and beverage companies on their nutrition-related policies and practices (Access to Nutrition Initiative, 2018). ATNI has a strong focus on investor engagement, and 72 investment organizations representing over USD$7 trillion assets under management had signed on to the ATNI investor statement by mid-2020 (Access to Nutrition Initiative, 2020). Signatories recognize that health and nutrition are key issues facing the food sector, and that companies able to anticipate and respond to these issues are better positioned to deliver financial performance (Access to Nutrition Initiative, 2020). Additionally, ShareAction, a charity based in the UK, are leading an initiative (ShareAction’s Healthy Markets Initiative) aimed at leveraging institutional investment to improve the obesity and nutrition related performance of UK food retailers and manufacturers (ShareAction, 2020). This includes improving policies and practices related to the healthiness and affordability of products, advertising of sugary products to children, and food labeling.

In Australia, a recent initiative from INFORMAS assessed the nutrition-related policies and commitments (including in relation to product composition, marketing to children, and food labeling) of the largest food and beverage manufacturers, food retailers and fast food companies in Australia (Sacks et al., 2018a,b,c). The methods for assessment were derived from ATNI, but were tailored to the local context and to each sector (Sacks et al., 2019b). While the initiative was not specifically directed at investors, the results provide Australian-specific data that could be used to inform corporate engagement and shareholder action activities. Research involving investment industry experts in Europe found that effective engagement activities need to include a business case for making changes and actionable demands that can be presented to companies (Eurosif, 2013). However, recent research from the UK indicates there are a number of barriers to shareholder action and engagement that aims to influence company practice and performance with regards to ESG (Ivanova, 2017). These include a misalignment of interests within the investment chain, a lack of transparency from companies on ESG issues, a lack of investor experience in shareholder action and engagement, conflicts of interest, diversified portfolios and a lack of resources; and limited demand for engagement from clients (Ivanova, 2017). The Australian Council of Superannuation Investors (ACSI), through their Stewardship Code, recommend that approaches to engagement should be publicly disclosed to facilitate greater accountability and good practice across the sector (Australian Council of Superannuation Investors, 2018). Future research should explore the barriers and enablers to shareholder action and engagement activities for ESG issues related to obesity and population nutrition specifically.

Only three of the institutional investors included in this review were signatories to the ATNI investor statement (AMP Capital, Local Government Super and Christian Super). AMP Capital and Local Government Super reported that they were engaged with the findings of the ATNI and were involved in corporate engagement regarding nutrition policies and practices of companies in the food sector. AMP Capital provided the most details of their corporate engagement and shareholder actions regarding obesity and population nutrition issues, reporting meeting with the boards and management of large Australian food and beverage companies to request disclosure around scientific funding and political donations, reducing marketing to children and young adults, reducing sugar content and reporting on sugar reduction targets. The engagement approach of AMP Capital was in line with recommendations from the ATNI, which highlights “products” (product formulation and nutrient profiling system), “marketing” (responsible marketing policy and auditing and compliance) and “engagement” (influencing governments, policy makers and stakeholder engagement) as three of seven key policy areas. Only one other investor, Christian Super, referred to marketing as part of their investment approach, specifically that they would be reviewing fast food companies on their marketing practices. Initiatives such as the ATNI and the ShareAction campaign can provide investors with a clear framework for engagement with food sector companies on improving their nutrition-related policies and practices. Outside of engagement and advocacy, these initiatives may be used to inform investment approaches that are based on the performance of food sector companies in relation to good practice benchmarks.

### Need for Comprehensive Reporting and ESG Data on Obesity and Population Nutrition Related Issues

While Corporate Social Responsibility reporting is now widespread (KPMG International, 2013, 2017), the lack of standardized metrics for reporting on obesity and population nutrition issues means that food sector companies are unlikely to be disclosing their nutrition-related policies and practices in a consistent and transparent way. The Sustainability Accounting Standards Board’s (SASB) (Sustainability Accounting Standards Board, 2020) reporting standards currently has “product health and nutrition” and “product labeling and marketing” metrics within their standards for the food and beverage, food retailing and restaurant sectors, although uptake and use is not yet widespread. The GRI previously had sector-specific standards (G4 Sector Disclosures) for the food processing sector (Global Reporting Initiative, 2019). Whilst reporting against these standards was voluntary, these sector-specific standards provided a framework for companies to report against policy and practice areas, such as product nutrition labeling, marketing communications (including marketing to children), product composition, programs and practices to promotion of healthy lifestyles, prevention of chronic disease, and access to healthy, nutritious and affordable food (Global Reporting Initiative, 2014). However, the G4 Sector Disclosures were superseded by the GRI Standards which do not include specific reporting indicators for nutrition-related policies and practices (Global Reporting Initiative, 2019). Furthermore, the variety of different ESG reporting initiatives and frameworks that currently exist, the various metrics and indicators employed across initiatives, and the voluntary nature of this reporting leads to significant heterogeneity in the ESG reporting of companies (Lokuwaduge and Heenetigala, 2017; Stubbs and Higgins, 2020). This heterogeneity may make it difficult for investors to receive comparable information on the obesity- and population nutrition-related ESG performance of food sector companies.

A number of investors included in this review noted that they used external ESG data providers to inform their decision making around investments, sometimes in addition to in-house ESG analytics. ESG data has previously been shown to lack comparability and consistency across providers, with significant variation in data sources and methodologies used (Wong et al., 2019). There are now also a large number of ESG data providers (more than 125 according to the Global Initiative for Sustainability Ratings), with some of the largest ESG data providers, such as RobecoSAM, MSCI, and Sustainalytics, all offering different data packages to investors (Kumar and Weiner, 2019). This further suggests that data on the ESG performance of companies provided to investors is likely to be highly variable. There is a need for comprehensive and consistent data on the performance of companies in the food sector that is widely available and can be used by investors in assessing performance related to obesity and population nutrition.

In Australia, whilst financial reporting for listed companies is regulated and required by law, ESG related disclosure rules are vague and form a minor component of overall disclosure requirements. Recommendation 7.4 of the Australian Stock Exchange (ASX) Corporate Governance Principles and Recommendations states that, “A listed entity should disclose whether it has any material exposure to environmental or social risks and if it does, how it manages or intends to manage those risks” (ASX Corporate Governance Council, 2019); however, there is limited detail on what this disclosure should include. Environmental and social risks mention “shortages of food;” however, they do not refer to direct risks related to obesity and nutrition. Australian standard-setters, such as the ASX Corporate Governance Council, could play a role in improving the comprehensiveness and consistency of disclosure related to ESG issues, including those related to obesity and population nutrition. There is also potential for the Australian Sustainable Finance Initiative, as a multi-stakeholder platform, to devise guidance and standards regarding these issues. This could include recommending disclosure of risks related to obesity and population nutrition in line with guidance from public health bodies such as the World Health Organization (World Health Organization, 2004) and benchmarking initiatives like the ATNI.

### Implications for Public Health

Institutional investors represent a potentially powerful vehicle for improving corporate governance and improving corporate practices in the food and beverage sector, as part of efforts to address key societal issues such as obesity and population nutrition. Encouragingly, the findings from this study indicate that investors are considering these issues to some extent, with some strong examples of how it is being addressed. However, there is currently substantial variation, and, for the most part, social issues related to obesity and population nutrition appear to be addressed in a minimal way within ESG. From a public health perspective, it is not clear what the most effective investment strategies (e.g., screening vs corporate engagement) and approaches (e.g., focusing on product portfolio versus policies and practices) to addressing issues related to obesity and population nutrition issues are likely to be. Nevertheless, it is likely that more comprehensive consideration of obesity and population nutrition issues, and clear statements of expectations from investors, will have a more substantial impact on food sector companies than current approaches. Selected application (e.g., by a small number of investors) of negative screening and divestment campaigns against unhealthy food sector companies may not be effective from a public health perspective as other investors may re-invest in their place. Positive screening and sustainability themed investments are promising in that they encourage investment in higher performing or healthier food sector companies; however that may have less of an impact on lower performing companies or those companies with less healthy portfolios, particular if the screening strategies are not applied by all investors. Corporate engagement and shareholder action activities may be an effective long-term option for improving the practices and performance of food sector companies, but this will rely on consensus around what is best practice for food companies and investors, consistent reporting (guided by clear reporting frameworks), detailed ESG data related to obesity and population nutrition, and use of standard nutrient profiling systems with respect to food sector companies.

In order to take advantage of increasing investor momentum around the SDGs (UN Global Compact, 2015; Dourma et al., 2017), there would appear to be value in building broader awareness for investors around the importance of addressing nutrition and obesity as part of achieving the SDGs by 2030. This could take various forms, such as highlighting the institutional investment case for addressing obesity and population nutrition as a key component of achieving a number of the SDGs. Explicit links to the SDGs could also be made by highlighting the co-benefits to environmental health of efforts to improve population nutrition. This could capitalize on the UN Decade of Action on Nutrition (2016 to 2025), of which Action Area 4 “Trade and investment for nutrition” mentions the need to invest responsibly in food systems (Food and Agricultural Organization of the United Nations, 2020).

### Strengths and Limitations

This study was the first to review major institutional investors in Australia on their approaches to incorporating ESG issues related to obesity and nutrition within their decision making. The desk-based review included a large sample of best practice institutional investors (36 asset managers and superannuation funds), which provides good insight into the current strategies being used by investors engaged in responsible investment in Australia.

A major limitation of this study was that it included only publicly available information, which may limit the comprehensiveness of data included in the study, with some investors not disclosing detailed responsible investment activities in the public domain. The desk-based approach was chosen primarily to manage the scope of this exploratory study, and is consistent with the approach taken by a number of other initiatives [such as the Business Benchmark on Animal Welfare (Amos et al., 2018), the Coller FAIRR Protein Producer Index (The FAIRR Initiative, 2019), and Plating up Progress (Nicholson, 2019)] that assess corporate practice and performance in regard to food system issues on the basis of publicly available information. Further research that draws on data from other sources, such as in-depth interviews and surveys, will enable better understanding of current practice and a more detailed assessment of leading investors in this area. Nevertheless, it is important that investors publicly disclose the bases on which they make responsible investment decisions, to facilitate good practice within the responsible investment community and to ensure key social issues like obesity and unhealthy diets are systematically considered. Moreover, a focus on publicly available information may encourage increased disclosure from corporations and enable corporations to be assessed in a consistent and objective way (Amos et al., 2018).

A second limitation of this study was that investment with regards to obesity and population nutrition issues was categorized into single responsible investment strategies for the purposes of reporting results. However, investors may apply multiple investment strategies sequentially or concurrently during investment and decision-making processes. Furthermore, many of the investors in this sample apply their approach to responsible investment in different ways for different funds. The desk-based nature of this study was not able to take these differences into account. Future research should involve in-depth discussion with the investment community to understand the investment strategies that are being applied in practice. It will be important to include a broad range of stakeholders in these discussions, including investors, ESG data providers, and regulatory bodies.

A third limitation was that the study did not evaluate investor strategies against the companies they actually invested in. There are likely to be a number of investors included in this sample that do not own food sector related assets, and, as such, issues related to obesity and nutrition are less relevant for these investors. Future analyses should explore the relationship between investors’ disclosed investment strategies and the companies in which they invest. This will also allow an analysis of the extent to which disclosed strategies are applied.

An additional area that warrants further exploration is investors’ motivations for consideration of obesity and population nutrition, and barriers and enablers to their inclusion within investment decision making processes. This should include international comparisons to investigate how the investment community is addressing these issues in other countries, and what key lessons there are for investors in Australia. In addition, it is recommended that other investors are investigated as part of future research in this area. Research from Clapp and colleagues (Clapp, 2019) indicates that a small number of large-scale asset managers (not those included in this study) are significant shareholders in some of the largest global food sector companies (Clapp, 2019). Engaging these and other large-scale global asset managers in discussion of ways to move toward healthier food systems is likely to be important and may hold the most potential for influence over companies in the food sector.

## Conclusion

There is significant potential for institutional investors to contribute to efforts to address obesity and improve population nutrition, as part of their approach to considering social considerations within ESG. The findings from this desk-based review indicate that a number of institutional investors in Australia are identifying ESG issues related to obesity and population nutrition as considerations, albeit in a limited way. The extent to which these considerations translate into investment decisions and the impact this has on companies in the food sector warrants further exploration. There is a need for future research that engages directly with investors to explore these uncertainties and identify opportunities for further progressing an investment agenda that contributes to efforts to address obesity and improve population nutrition in Australia.

## Data Availability Statement

The original contributions presented in the study are included in the article/Supplementary Materials, further inquiries can be directed to the corresponding author/s.

## Author Contributions

ER, CP, RC, and GS were involved in the conceptualization and design of the study. ER conducted the data collection and data analysis and drafted the manuscript. CP and GS were involved in cross-checking data. All authors reviewed and edited the manuscript.

## Conflict of Interest

The authors declare that the research was conducted in the absence of any commercial or financial relationships that could be construed as a potential conflict of interest.

## Footnotes

1 One of the asset managers identified in the 2019 RIAA Responsible Investment Benchmark Report, JCP Investment Partners, was no longer operational at the time the study was conducted and, as such, was excluded. One other asset manager identified in the 2019 RIAA Responsible Investment Benchmark Report, New Forests, invested exclusively in forestry real assets and was also excluded because of the highly focused nature of their portfolio with limited relevance to the topic of the study.

2 There was no useable list of largest funds in the 2019 benchmark report, so this group could not be updated for 2019.

3 One superannuation fund identified in the 2019 RIAA Responsible Investment Super Study, Future Fund, was excluded due to it being a sovereign wealth fund, and, therefore, was considered to have different strategic and operational requirements to the other funds included in the sample. NZ Super Fund was excluded because it is not based in Australia.

4 Australian superannuation funds were not included in the scope of the 2019 RIAA Responsible Investment Benchmark Report, with the exception of Australian Ethical. Australian Ethical was already included in the first group of asset managers and, as such, was only counted once in the overall total of included investors (*n* = 35).

5 INFORMAS is a global network that aims to monitor, benchmark, and support public- and private-sector actions to create healthy food environments Swinburn et al. (2013). INFORMAS (International Network for Food and Obesity/non-communicable diseases Research, Monitoring and Action Support): overview and key principles. *Obesity Reviews* (S1), 1.

6 Australian Ethical was included in both the asset manager and superannuation fund sample.
