# Supplementary material for: The Extent to Which Obesity and Population Nutrition Are Considered by Institutional Investors Engaged in Responsible Investment in Australia - A Review of Policies and Commitments
Source: Front Psychol. 2020 Dec 23;11:577816. doi: 10.3389/fpsyg.2020.577816 (PMC7793752; doi:10.3389/fpsyg.2020.577816)
Supplement: Supplementary file 1 [file Table_1.DOCX]

Supplementary Material

**Table S1: Responsible investment strategy and criteria for coding of strategies**

| **Responsible investment strategy** | **Criteria for coding of strategies** |
| --- | --- |
| 1. Negative/exclusionary screening | - Specifically referred to as ‘negative or exclusionary screening’ - Screening that applies an exclusion to specific types of companies or sectors |
| 2. Positive/best-in-class screening | - Specifically referred to as ‘positive’ or ‘best-in-class’ screening - Performance compared to peers but using a defined method (e.g., through a benchmark) |
| 3. Norms-based screening | - Specifically referred to as ‘norms-based screening’ - Refers to an international convention, framework or body in relation to screening strategy |
| 4. ESG integration | - Specifically referred to as ‘integration of ESG factors’ or ‘ESG integration’ - Discussion of risks and opportunities in relation to ESG issues - Inclusion of ESG issues into the decision-making process |
| 5. Sustainability-themed investing | - Refers specifically to ‘sustainability-themed’ investments - Particular fund or strategy where investments are targeted towards sustainability-themed companies or industries, or solves particular sustainability challenges |
| 6. Impact and community investing | - Refers specifically to impact on a community or a particular cause - Investment in specific community projects |
| 7. Corporate engagement and shareholder action | - Engagement with companies - Proxy voting - Advocacy directed at company management |

ESG = Environmental, Social, Governance
