# Supplementary material for: The Extent to Which Obesity and Population Nutrition Are Considered by Institutional Investors Engaged in Responsible Investment in Australia - A Review of Policies and Commitments
Source: Front Psychol. 2020 Dec 23;11:577816. doi: 10.3389/fpsyg.2020.577816 (PMC7793752; doi:10.3389/fpsyg.2020.577816)
Supplement: Supplementary file 2 [file Table_2.DOCX]

Supplementary Material

**Table S2: Obesity and population nutrition themes, sub-themes and definitions**

| **Theme** | **Sub-theme** | **Definition** |
| --- | --- | --- |
| General health considerations relevant to obesity and population nutrition | Health (broadly defined) | Considerations related to investments impact (positive or negative) on health (e.g., companies or products that improve community health and wellbeing, considering ‘health’ as an ESG issue area). Does not include tobacco or alcohol related considerations. |
|  | Obesity and nutrition (broadly defined) | Considerations that explicitly mention obesity and/or nutrition. |
|  | SDG2: Zero hunger | Considerations that specifically mention SDG2.  SDG2 = End hunger, achieve food security and improved nutrition and promote sustainable agriculture^1^. |
|  | SDG3: Good health and wellbeing | Considerations that specifically mention SDG3.  SDG3 = Ensure healthy lives and promote wellbeing for all at all ages^1^. |
| Company nutrition policies and practices | General policies and practices related to obesity and population nutrition | Refers to strengthening nutrition-related policies and practices (in general, not specific to a particular policy/practice area). |
|  | Food marketing | Refers to companies advertising policies and marketing practices. |
|  | Food reformulation/product development | Refers to improving nutritional qualities of food and beverage products, including reducing sugar and salt content. |
|  | Disclosure and transparency around relationships with external groups | Refers to improved disclosure of political donations and scientific research funding by food sector companies. |
| Company product portfolio | Companies or products that are 'healthy' | Considerations related to the ‘healthiness’ of the product portfolio of food sector companies, with a focus on food categories or products considered ‘healthy’, e.g. fruit and vegetables |
|  | Companies or products that are 'unhealthy' | Considerations related to the ‘healthiness’ of the product portfolio of food sector companies, with a focus on food categories or products considered ‘unhealthy’ e.g. sugary drinks, confectionery |
|  | Diversification of earnings stream | Refers to diversification of earnings streams e.g. through a focus on multiple product categories and market sectors. |

SDG = Sustainable Development Goal

^1^Sourced from: United Nations. *Sustainable Development Goals*. United Nations; 2015. Available at: <http://www.un.org/sustainabledevelopment/sustainable-development-goals/>.
