# Supplementary material for: The Extent to Which Obesity and Population Nutrition Are Considered by Institutional Investors Engaged in Responsible Investment in Australia - A Review of Policies and Commitments
Source: Front Psychol. 2020 Dec 23;11:577816. doi: 10.3389/fpsyg.2020.577816 (PMC7793752; doi:10.3389/fpsyg.2020.577816)
Supplement: Supplementary file 3 [file Table_3.DOCX]

Supplementary Material

**Table S3: Number of responsible investment strategies related to obesity and population nutrition disclosed by asset managers and superannuation funds**

| Asset manager/  superannuation fund | Negative/  exclusionary screening | Positive/  best-in-class screening | ESG integration | Sustainability-themed investing | Corporate engagement and shareholder action | Total number of strategies |
| --- | --- | --- | --- | --- | --- | --- |
| Australian Ethical | ✓ | ✓ | ✓ |  |  | 3 |
| Stewart Investors |  |  | ✓ | ✓ | ✓ | 3 |
| AMP Capital |  |  | ✓ |  | ✓ | 2 |
| Christian Super | ✓ |  | ✓ |  |  | 2 |
| First Sentier Investors |  |  | ✓ | ✓ |  | 2 |
| Pendal |  | ✓ | ✓ |  |  | 2 |
| U Ethical |  | ✓ |  | ✓ |  | 2 |
| Pengana Capital |  |  |  | ✓ |  | 1 |
| Magellan Asset Management |  |  | ✓ |  |  | 1 |
| Ausbil Investment Management |  |  | ✓ |  |  | 1 |
| CareSuper |  |  |  | ✓ |  | 1 |
| Future Super |  | ✓ |  |  |  | 1 |
| HESTA |  |  | ✓ |  |  | 1 |
| Local Government Super |  |  |  |  | ✓ | 1 |
| Mercer Australia |  |  | ✓ |  |  | 1 |
| Perpetual Investments |  |  | ✓ |  |  | 1 |
| Stafford Capital Partners |  |  | ✓ |  |  | 1 |
| Uniting Financial Services |  |  |  | ✓ |  | 1 |
| *AustralianSuper, Cbus, Dexus Property Group, First State Super, IFM Investors, Investa Property Group, Lendlease Investment Management, Maple-Brown Abbot, QIC, RARE Infrastructure, Realindex Investments, Resolution Capital, Solaris Investment Management, Unisuper, VicSuper, Vision Super, Wavestone Capital* | | | | | | 0 |
