# Supplementary material for: The Extent to Which Obesity and Population Nutrition Are Considered by Institutional Investors Engaged in Responsible Investment in Australia - A Review of Policies and Commitments
Source: Front Psychol. 2020 Dec 23;11:577816. doi: 10.3389/fpsyg.2020.577816 (PMC7793752; doi:10.3389/fpsyg.2020.577816)
Supplement: Supplementary file 4 [file Table_4.DOCX]

Supplementary Material

**Table S4: Details of investment strategies of asset managers and superannuation funds and the obesity/population nutrition theme to which they relate**

| 1. **Negative/exclusionary screening** | | | | |
| --- | --- | --- | --- | --- |
| **Theme: Health considerations relevant to obesity and population nutrition** | | | | |
| Asset manager/  superannuation fund | Sub-theme | | | Details |
| Australian Ethical | Health considerations (broadly defined) | | | - - - - “Australian Ethical shall avoid any investment which is considered to unnecessarily: extract, create, produce, manufacture, or market materials, products, goods or services which have a harmful effect on humans, non-human animals or the environment”       - **PRI Transparency report 2019** Strategy and governance >> Objectives and strategies >> SG 06. Main goals/objectives this year   “Screening of all investments under the ‘Australian Ethical Charter’, taking account of positive and negative impacts on people, animals and environment.  *Progress achieved*  All potential new investments were assessed under our Ethical Charter. We did not invest in 'non-aligned' companies (other than nominal holdings held for advocacy purposes).” |
| **Theme: Company product portfolio** | | | | |
| Asset manager/  superannuation fund | Sub-theme | | | Details |
| Australian Ethical | Companies or products that are ‘unhealthy’ | | | - “HEALTHY DIET CRITERIA   Food must be nutritious before we can invest in its production. In assessing whether a food can form part of a balanced and healthy diet we take into account credible sources like the World Health Organisation’s (WHO) healthy diet guidelines. In practice, this means we are unlikely to invest in producers of food that is overconsumed (eg, sugar) and look more favourably on producers of fruits, vegetables, legumes, nuts and whole grains. On the processing side, we avoid foods that are high in trans-fats, sugar or salt (in line with the WHO guidelines).” |
| Christian Super | Companies or products that are ‘unhealthy’ | | | - “We actively avoid companies that:   - Produce addictive or harmful goods or services     - - - Excessively damage the environment         - Utilise exploitation or predatory practices         - Seriously violate human rights   In industries like:   - - - - - Gambling         - Pornography         - Weapons         - Alcohol & tobacco         - Child labour         - Embryonic stem cell research         - Fast food” |
| 1. **Positive/best-in-class screening** | | | | |
| **Theme: Health considerations relevant to obesity and population nutrition** | | | | |
| Asset manager/  superannuation fund | Sub-theme | | | Details |
| Pendal | Health considerations (broadly defined) | | | - “Our positive screens includes industries with products and services that provides a direct benefit to social and/or environmental outcomes such as: environmental management or remediation environmental technologies, energy efficiency and renewable energy, low-impact products or products that reduce ecological footprint, sustainable buildings and property development, sustainable land use and food production, improved health and community well-being.” |
| UEthical | Health considerations (broadly defined) | | | - **PRI Transparency report 2019** Direct - Listed Equity Incorporation » ESG incorporation in actively managed listed equities » Implementation processes » (A) Implementation: Screening   “Our ethical investment policy and positive screening encourages investment in companies that produce goods or services which: Enhance the health and welfare of individuals and communities….” |
| **Theme: Company product portfolio** | | | | |
| Asset manager/  superannuation fund | Sub-theme | | | Details |
| Australian Ethical | Companies or products that are ‘healthy’ | | | - “…food has to meet three criteria to be investable under our charter. Specifically, food must: Form part of a healthy diet..” - “HEALTHY DIET CRITERIA   Food must be nutritious before we can invest in its production. In assessing whether a food can form part of a balanced and healthy diet we take into account credible sources like the World Health Organisation’s (WHO) healthy diet guidelines. In practice, this means we are unlikely to invest in producers of food that is overconsumed (eg, sugar) and look more favourably on producers of fruits, vegetables, legumes, nuts and whole grains. On the processing side, we avoid foods that are high in trans-fats, sugar or salt (in line with the WHO guidelines).” |
| Future Super | Companies or products that are ‘healthy’ | | | - **“Approved investments -** Our positive screening process actively seeks out companies involved in the following activities:   - ……….Production of healthy foods - Production of healthy foods and support for healthy lifestyles   - Social wellbeing - Activities that promote dignity and wellbeing, and alleviate poverty…..” |
| 1. **Norms-based screening** | | | | |
| No details identified. | | | | |
| 1. **ESG integration** | | | | |
| **Theme: Health considerations relevant to obesity and population nutrition** | | | | |
| Asset manager/  superannuation fund | Sub-theme | | | Details |
| HESTA | Health considerations (broadly defined) | | | - “Looking at an investments impact on people and planet helps us to more fully understand its risks and opportunities, particularly over the longer term. Considerations when making decisions include:   - “Good health and wellbeing…..” |
| Magellan Asset Management | Health considerations (broadly defined) | | | - **PRI Transparency report 2019** Direct - Listed Equity Incorporation » ESG incorporation in actively managed listed equities » Implementation processes » (A) Implementation: Screening. LEI 04. Types of screening applied   “Magellan believes that companies engaging in business that undermines the rule of law or that have major detrimental impacts on human health warrant particularly close scrutiny by investors. Such business models may be the subject of future government regulation or changes in consumption patterns, making them a high risk investment from investors’ perspective.” |
| Mercer Australia | Health considerations (broadly defined) | | | - “From an ESG perspective, all of the long-term trends listed here have a significant impact on the demand drivers for sustainability-themed investments - i.e. demographics, energy and resource demand/use and technology - as they are all linked to climate change in particular, but also broader ESG trends and investment themes, which include education, health, waste, water etc. Mercer's ongoing intellectual capital work - from within the RI team and more broadly across the firm (particularly the MMC Insights Center) - explores all of these issues and is reflected in our investment advice, manager research (on the investment strategies available to clients), issue specific research (e.g. our climate change reports) and in how we monitor client portfolios.” |
| Pendal | Health considerations (broadly defined) | | | - **PRI Transparency report 2019** Direct - Fixed Income » ESG incorporation in actively managed fixed income » (C) Implementation: Integration>>FI 12. Integration – E,S and G issues reviewed. FI 12.2. Please provide more detail on how you review E, S and/or G factors in your integration process.   “ESG factors are typically captured in the business profile and risk factor categories where they are deemed material to the financial outcomes of the fund using bottom-up analysis. Examples of some of the categories that may be considered (depending on specific sector, business factors, investment horizon, geographic exposures, regulatory factors, and product performance objectives) include…… • [Social practices] products or services that provide positive social impacts such as improved health & community well-being, disease prevention, and education” |
| Perpetual Investments | Obesity/nutrition considerations | | | - - - - “Perpetual’s Ethical SRI strategy has two screens, including: An immediate exclusion for involvement in certain industries through the implementation of a 5% revenue materiality threshold; and a performance screen based on an evaluation of companies’ overall sustainability performance on SRI criteria….. Companies are scored (positively or negatively) on their approach to or involvement in the SRI issues shown in the following table:   Social SRI Issue: Obesity” [one of several issues listed] |
| Stafford Capital Partners | SDG3: Good Health and Wellbeing | | | - “Stafford has been actively incorporating SDGs across the organisation… At the time of writing, we have reviewed 550 companies for SDG alignment in terms of general business activity. Companies range from an alignment to zero SDG targets while other companies may align to as many as eight….Across the portfolio, all 17 SDGs were present, however, there was a clear weighting towards a few SDGs. SDGs 9 – Innovation and Infrastructure, 3 – Good Health and Well Being, 8 – Decent Work and Economic Development, 7 – Affordable and Clean Energy, 12 – Responsible Consumption and Production, and 11 – Sustainable Cities and Communities account for over 86% of the total SDG alignment.” [Stafford’s list of SDG targets applicable to private equity companies includes SDG2 and SDG3] - “The SDG review of individual companies will be conducted on all potential investment opportunities considered for Stafford Sustainable Capital Fund 3… the SDG review is incorporated into the investment decision process for every potential investment considered by the fund.” |
|  | SDG2: Zero Hunger | | |  |
| **Theme: Company nutrition policies and practices** | | | | |
| Asset manager/  superannuation fund | Sub-theme | | | Details |
| Christian Super | Food marketing | | | - “Christian Super places a focus on sectors and companies that supply food which contribute to the problems of unhealthy eating by providing nutritionally empty foods, by not providing healthy alternatives in their product range, and by manipulating their food content to encourage excessive and unhealthy levels of consumption. Christian Super will review major food (including fast food) producers to assess their overall commitment to nutrition and health through their product range, marketing practices and promotion of active lifestyle, with a view of excluding those companies that consistently perform below industry standards.” |
| **Theme: Company product portfolio** | | | | |
| Asset manager/  superannuation fund | Sub-theme | | | Details |
| AMP Capital | Companies or products that are ‘unhealthy’ | | | - "The investment impacts of obesity are far reaching, with the rising associated healthcare costs – particularly in relation to type 2 diabetes – steering governments towards the introduction of sugar/soda taxes or similar disincentives to curb sugar consumption. These regulatory interventions, combined with the burgeoning wellness trend, pose a significant risk to the earnings of a large number of food and beverage companies if they fail to act" |
|  | Companies or products that are ‘unhealthy’ | | | - "There’s a good chance that meat will be taxed in the next five to ten years in an effort to fight global challenges such as climate change, deforestation, antibiotic resistance and obesity. In terms of its health effects, meat consumption is linked to an increasing incidence of global obesity and associated type 2 diabetes and cancer.... While meat taxation is not a short-term risk for investors, large pension funds and asset managers should be thinking about these issues" |
| Ausbil Investment Management | Companies or products that are ‘unhealthy’ | | | - "The ‘obesity’ theme is well documented and presents both opportunities and risks for investors…. there are food & beverage companies that are negatively exposed and with limited possibility for adaptation to changing consumer and regulatory trends, for which it is hard to see an easy transition" |
| Australian Ethical | Companies or products that are ‘unhealthy’ | | | - “Food production plays an essential role in the well-being of people and alleviation of poverty (important focuses for our ethical impact). But farming can also have negative impacts on people, animals and the environment. When considering agricultural activities and companies we look at:   - health impacts of the food produced; …..” |
|  | Companies or products that are ‘healthy’ | | |  |
| First Sentier Investors | Companies or products that are ‘unhealthy’ | | | - “PEP [PepsiCo] is also exposed to nutrition-related issues regarding its snack-foods (high salt, high sugar, etc.) as well as its soft drink products which are increasingly targeted by anti-obesity groups in the states (as well as anti high-fructose corn syrup headlines). Carbonated soft drink consumption in the U.S. continues to be on a long-term gradual decline, at least in part due to public health concerns” |
| Christian Super | Companies or products that are ‘unhealthy’ | | | - “Christian Super places a focus on sectors and companies that supply food which contribute to the problems of unhealthy eating by providing nutritionally empty foods, by not providing healthy alternatives in their product range, and by manipulating their food content to encourage excessive and unhealthy levels of consumption. Christian Super will review major food (including fast food) producers to assess their overall commitment to nutrition and health through their product range, marketing practices and promotion of active lifestyle, with a view of excluding those companies that consistently perform below industry standards.” |
| Magellan Asset Management | Companies or products that are ‘unhealthy’ | | | - “It’s a campaign that targets sugar – fat to a lesser extent – and one that undermines the sales growth of sugary food and beverage Big Brands…. Big Brands are responding by reducing sugar content, launching healthier products, expanding into non-sugary businesses and substituting sugar with artificial sweeteners, though this switch is backfiring because sweeteners are proving to be health risks. The campaign to reduce the obesity that has been a health issue since the 1970s will threaten processed-food companies for the foreseeable future” |
| Pendal | Companies or products that are ‘unhealthy’ | | | - “Coca Cola Amatil (CCL) is one of Asia-Pacific’s largest bottlers and distributors of alcoholic and non-alcoholic beverages. The majority of its products are non-alcoholic and high in sugar. For many years, Pendal Group Limited (Pendal) has held concerns regarding headwinds from structural shifts in consumer demand for healthier options and regulatory risks relating to sugar consumption and their associated impacts on corporate profitability. Pendal has held an underweight position in CCL across its Australian fixed income funds for a number of years, given these concerns…… Pendal’s position on the company reflects its view that the social risks around high sugar and its links to diabetes and obesity have not been priced in to the issuer’s credit spread and hence Pendal expected CCL’s credit spreads to underperform over time. There are also regulatory risks surrounding potential imposition of sugar taxes in key markets. From a financial perspective, CCL’s credit spreads have been tight and, in Pendal’s view, have not factored in social risks relative to similarly rated issuers” |
| Stewart Investors | Companies or products that are ‘unhealthy’ | | | - - - - “Over the quarter, we sold out of CT Holdings as we believe many of its underlying businesses will face sustainability headwinds longer term due to a rising exposure to unhealthy foods through some of their own brands and franchise partnerships with KFC and TGIF.”       - “Asia’s obesity epidemic will affect the profit that can be generated from selling products with high sugar, fat and salt content over a period of years rather than months, as a combination of government regulation and changing consumer preferences slowly takes hold” |
|  | Companies or products that are ‘healthy’ | | | - - - - “Significant portfolio changes: In addition we added to Robinsons Retail as they remain focused on providing fresh and healthy products at reasonable prices throughout the Philippines”       - “Asia Pacific Leaders acquired Uni-President Enterprise (UPE) during the quarter. UPE was founded as a humble flour mill in the 1960s and has built strong market shares in the food and beverage markets in Taiwan and China. The group is carefully stewarded by the Lo family who have repeatedly shown themselves to be extremely high quality, as exhibited by an increasing focus on the provision of healthier products.” |
| 1. **Sustainability–themed investing** | | | | |
| **Theme: Health considerations relevant to obesity and population nutrition** | | | | |
| Asset manager/  superannuation fund | Sub-theme | | | Details |
| CareSuper | Obesity/nutrition theme (broadly defined) | | | - - - - “Positive themes (Overseas and Australian shares)   We seek out positive investments that help improve the environment and the quality of our daily life such as:   - - - - - Access to safe food and water – sustainable food systems, improved nutrition and improve water equipment and services….” |
|  | SDG3: Good Health and Wellbeing | | | - - - - Aligning with the UN Sustainable Development Goals – “With our ongoing process of researching, analysing and monitoring investments, we ensure high ESG standards are being met. We’re not only assessing outcomes against our own benchmarks – our investment managers also choose investments that align with the United Nation’s Sustainable Development Goals in a meaningful way. These goals have a global reach, aiming to combat issues including poverty, inequality and environmental degradation.         - Our SBO supports a number of the United Nations Sustainable Development Goals.         - Goal 1 – No poverty         - Goal 3 – Good health         - Goal 4 – Quality education         - Goal 6 – Clean water and sanitation         - Goal 9 – Industry, innovation and infrastructure         - Goal 11 – Sustainable cities and communities         - Goal 12 – Responsible consumption         - Goal 13 – Climate action” |
| First Sentier Investors | Health theme (broadly defined) | | | - - - **PRI Transparency report 2019** Direct - Listed Equity Incorporation » ESG incorporation in actively managed listed equities » Implementation processes » (B) Implementation: Thematic. LEI 07. 07.2. Describe your organisation’s processes relating to sustainability themed funds.   “The sustainability strategies augment this process by seeking to invest in the shares of those companies which the team believes are particularly well positioned to benefit from, and contribute to, the sustainable development of the countries in which they operate. The team assesses sustainability through three lens:   1. Sustainable Goods and Services    - Companies with a positive impact on society, environment, health    - Well positioned for shifting consumer preferences, regulatory headwinds, long-tail liabilities” |
| Pengana Capital | Health theme (broadly defined) | | | - - - - Pengana WHEB Sustainable Impact Fund “…WHEB have identified critical environmental and social challenges facing the global population over the coming decades. WHEB invests in a diverse portfolio of global companies providing solutions to these sustainability challenges via nine sustainable investment themes – five of these are environmental (cleaner energy, environmental services, resource efficiency, sustainable transport and water management) and four are social (education, health, safety and well-being).” |
| UEthical | SDG2: Zero Hunger | | | - - - - “Investing ethically and for impact [SDG2]… Many, even in developed world countries, are at risk of living in hunger, with lack of access to affordable nutritious food. This issue affects at least 5 per cent of Australians, with at-risk groups including the unemployed, single parent households and young people. Nutrition, hygiene and personal care company Unilever derives more than half its revenue from provision of nutrition and sanitation products and in recent years has heavily invested in sustainable supply improvements. The A2 Milk Company provides access to A2 beta casein protein milk products. This protein has been shown to provide benefits to those with lactose intolerance. As at 31 December 2017, both companies were included in our portfolios.” |
| Uniting Financial Services | Health theme (broadly defined) | | | - **PRI Transparency report 2019** Direct - Fixed Income » ESG incorporation in actively managed fixed income » (A) Implementation: Screening   “In addition to this we are actively looking for organisations that have a positive impact. More specifically we are targeting green bonds, climate related RMBS and social impact related issues. Other activities will be favourably regarded which accomplish outcomes such as:   - improving human health, dignity and well-being;….” - “The Church also wishes to encourage positive activities, where doing so is also sound from an investment perspective. Activities will be favourably regarded which accomplish outcomes such as:   • reducing human suffering; • improving human health, dignity and well-being; • eradication of unethical practices; • amelioration of pollution or other environmental damage; or the development of sustainable buildings, practices, etc” |
| **Theme: Company product portfolio** | | | | |
| Asset manager/  superannuation fund | Sub-theme | | | Details |
| Pengana Capital | Companies or products that are ‘unhealthy’ | | | - - - - [Observations from Kraft Heinz’s attempted takeover of Unilever] “To declare our position upfront, WHEB [sustainable impact fund] wouldn’t actually invest in either. Both of these companies sell consumer staples: household products, and packaged food. Neither of those categories solve a sustainability challenge. Indeed, some of their brands contribute to unhealthy lifestyles. I’m looking at you, Oscar Mayer hotdogs (Kraft), and you, Ben and Jerry’s ice cream (Unilever).” |
| Stewart Investors | Companies or products that are ‘healthy’ | | | - - - - “We find it useful as a first step to classify potential investment opportunities … 1. Sustainable goods and services: We seek out companies that:         - Provide food, beverages and consumer staples that are positive for human health and hygiene, affordable medicines and life-saving treatments, and other products, technologies and services that contribute to economic development and human welfare and safety;         - Manufacture, sell and deliver products and services efficiently and in ways that reduce negative environmental and social impacts; and         - Stand to benefit as environmental and social externalities are internalised         - Examples of such companies include Marico Ltd (India: healthier foods that reduce the risk of diabetes), CSL Ltd (Australia: vaccines and plasma protein), Henkel AG & Co KGaA (Germany: consumer and industrial adhesives), Kikkoman (Japan: naturally fermented soy products, beverages and seasonings) and Unilever (UK: producer of consumer staples).”       - “The private sector, by virtue of its scope and pervasiveness, has a vital role to play in developing a sustainable solution to the problem of malnutrition. As such, we continue to aim to allocate clients’ capital to companies that we feel are beginning to realise the necessity improving access to nutritious products, and believe that this is beneficial both for society and long-term investment returns”       - “The sustainability headwinds that malnutrition produces for companies over a long-time horizon, be it in the form of an increased tax on high sugar, high calorie products or changing patterns of consumption, are clearly very real. The Mexican government implemented a roughly 10% nationwide tax on sugar-sweetened beverages in January 2014. Since then, the sales of sugary drinks have fallen by 5.5% in 2014 and by 9.7% in 2015… The private sector, by virtue of its scope and permeation, has a vital role to play in developing a sustainable solution to the problem of malnutrition. As such, we continue to aim to allocate clients’ capital to companies that we feel are beginning to realise the magnitude of the challenge that lies ahead, and hope to support management in the journey to improving access to nutrition.” |
| 1. **Impact/community investing** | | | | |
| No details identified. | | | | |
| 1. **Corporate engagement and shareholder action** | | | | |
| **Theme: Company nutrition policies and practices** | | | | |
| Asset manager/  superannuation fund | | Sub-theme | Details | |
| AMP Capital | | General policies and practices related to obesity and population nutrition | - “In February, AMP Capital joined the ATNI Investor Engagement Group, which will see AMP Capital take part in collective engagement with 17 listed companies within the 2018 Global ATNI, the third Index of the series, due to be launched later this year. Engagement will focus on each company’s approach to integrating ATNI’s [Access to Nutrition Index] recommendations and more generally improving nutrition-related policies and practices.” | |
|  |  | Food marketing | - “AMP Capital has been meeting with the boards and management of Australia’s largest food and beverage manufacturers asking for … changes to the way the companies advertise to children. AMP capital has been asking food and beverage companies … for details of advertising policies…”. - “AMP Capital is actively researching the earnings risks posed by exposure to sugar and engaging with Australian food and beverage manufacturers and retailers on the need to… reduce advertising to children and young adults.” | |
|  |  | Food reformulation/product development | - “AMP Capital has been meeting with the boards and management of Australia’s largest food and beverage manufacturers asking for reductions in sugar usage…. AMP capital has been asking food and beverage companies to report on their progress meeting sugar reduction targets…” - “AMP Capital is actively researching the earnings risks posed by exposure to sugar and engaging with Australian food and beverage manufacturers and retailers on the need to diversify earnings streams, reduce sugar content…” | |
|  |  | Disclosure and transparency around relationships with external groups | - “AMP capital has been asking food and beverage companies to report on their progress meeting sugar reduction targets as well as for details of advertising policies and information about how they fund scientific research.” - “What we are calling for: Lower sugar content in packaged products; Reduced advertising to children and young adults; Clear disclosures on political donations and the funding of scientific research on sugars impact on human health and obesity”. | |
| Local Government Super | | General policies and practices related to obesity and population nutrition | - **PRI Transparency report 2019** Direct - Listed Equity Active Ownership » Outputs and outcomes>LEA 11. Examples of ESG engagements   “To increase company awareness of the Access to Nutrition Index 2016 findings and ask how companies are integrating these recommendations into improving the nutritional benefits of their products. This was under the belief that companies that are applying strong nutrition policies and practices globally are better positioned to reduce the risk of increasing food and beverage industry regulation and to take full advantage of changing consumer trends towards healthier living.”  “A letter was drafted by four lead investors to 13 multinational food companies. ATNI signatories and other international investors were then asked to co-sign the letter before it was sent to these companies.” | |
|  |  | Food reformulation/product development | - **PRI Transparency report 2019** Direct - Listed Equity Active Ownership » Outputs and outcomes>LEA 11. Examples of ESG engagements   “To increase company awareness of the Access to Nutrition Index 2016 findings and ask how companies are integrating these recommendations into improving the nutritional benefits of their products. This was under the belief that companies that are applying strong nutrition policies and practices globally are better positioned to reduce the risk of increasing food and beverage industry regulation and to take full advantage of changing consumer trends towards healthier living.”  “A letter was drafted by four lead investors to 13 multinational food companies. ATNI signatories and other international investors were then asked to co-sign the letter before it was sent to these companies.” | |
| Stewart Investors | | Food reformulation/product development | - “During the quarter for the Asia Leaders Strategy we initiated positions in Vitasoy, Kasikornbank and Pigeon Corporation….. Vitasoy provides high-quality soya-based beverages and has a dominant position in HK and profitable growth in China and the Philippines. We have invested in Vitasoy for many years in our all cap portfolios and have a lot of admiration for the Lo family who founded the business over eighty years ago. The group embraces sustainability but we continue to engage with the stewards on sugar content and package wastage.” | |
| **Theme: Company product portfolio** | | | | |
| Asset manager/  superannuation fund | | Sub-theme | Details | |
| AMP Capital | | Diversification of earnings stream | - “AMP Capital is actively researching the earnings risks posed by exposure to sugar and engaging with Australian food and beverage manufacturers and retailers on the need to diversify earnings streams, reduce sugar content, and reduce advertising to children and young adults.” | |

SDG = Sustainable Development Goal
